# Cataract surgery in eyes with adult-onset foveomacular-vitelliform dystrophy

**DOI:** 10.1007/s00417-025-07018-x

**Published:** 2025-11-03

**Authors:** Or Shmueli, Tomer Batash, Itay Nitzan, Itay Chowers, Liran Tiosano

**Affiliations:** Ophthalmology Department, Faculty of Medicine, Hadassah Medical Center, Hebrew University, POB 12000, Jerusalem, 91120 Israel

**Keywords:** Adult-Onset Foveomacular-Vitelliform dystrophy, Cataract surgery, Outcomes, Efficacy, Safety

## Abstract

**Purpose:**

To assess the outcomes and safety of cataract surgery in Adult-Onset Foveomacular-Vitelliform dystrophy (AFVD) patients.

**Methods:**

This retrospective study analyzed eyes with AFVD that underwent cataract surgery in a tertiary center, comparing them with eyes affected by none and neovascular age-related macular degeneration (NVAMD). Data included demographics, best-corrected visual acuity LogMAR (VA), eye examination results, and optical coherence tomography (OCT) results. The primary outcome was improvement in VA. A secondary outcome was AFVD progression such as the development of choroidal neovascularization (CNV) or retinal atrophy.

**Results:**

The cohort included 83 eyes (18 with AFVD, 27 with none-NV AMD, and 38 with NVAMD). AFVD eyes showed significant improvement in VA±SD from pre-surgery (0.60 ± 0.34) to 1 month (0.23 ± 0.11; *P* < 0.0001) and 12 months post-operatively (0.26 ± 0.12; *P* < 0.0001). No cases of CNV or major AFVD progression changes were observed over 12 months of follow up. However, at last follow-up (62.20 ± 39.30 months) there was increased proportion of atrophic AFVD (44.40% compared to 5.50% at baseline; *P* = 0.10).

No difference was found comparing VA improvement one month after surgery of none-NV AMD, NVAMD and AFVD (AFVD = 0.37 ± 0.36, none-NV AMD = 0.47 ± 0.68, NVAMD = 0.28 ± 0.37; *P* = 0.29). In the none-NV AMD group, one eye developed CNV ten months post-operatively and another eye demonstrated worsening retinal atrophy one-month post-surgery. In the NVAMD group, 9 eyes developed retinal atrophy at last follow up.

**Conclusion:**

Cataract surgery in AFVD eyes led to significant visual acuity improvement and demonstrated good safety with no new CNV or retinal atrophy. The similar visual improvement across the AFVD, none-NV AMD and NVAMD groups suggests the procedure’s efficacy and safety for AFVD patients.

**Supplementary Information:**

The online version contains supplementary material available at 10.1007/s00417-025-07018-x.

## Key messages

*****Known key messages***:**

- Studies on Age-related macular degeneration (AMD) indicate minimal progression post-cataract surgery.
- However, the safety of cataract surgery in patients with coexisting Adult-onset foveal-macular vitelliform dystrophy (AFVD) remain unexplored.

*****New key messages***:**

- The study reports on the outcomes of cataract surgery in AVFD eyes.
- Significant visual acuity improvements were seen with no significant complications, demonstrating good safety and efficacy of cataract surgery in AFVD eyes.

**Supplementary Information:**

The online version contains supplementary material available at 10.1007/s00417-025-07018-x.

## Introduction

Adult-onset foveal-macular vitelliform dystrophy (AFVD), first reported by Gass in 1974, is characterized by a yellow, solitary, round, or oval subretinal macular lesion similliar to but typically smaller than that observed in juvenile-onset Best’s disease [1].

The condition is thought to result from retinal pigment epithelium (RPE) dysfunction, leading to the accumulation of yellowish material beneath the neurosensory retina [2].

**Fig. 1:**
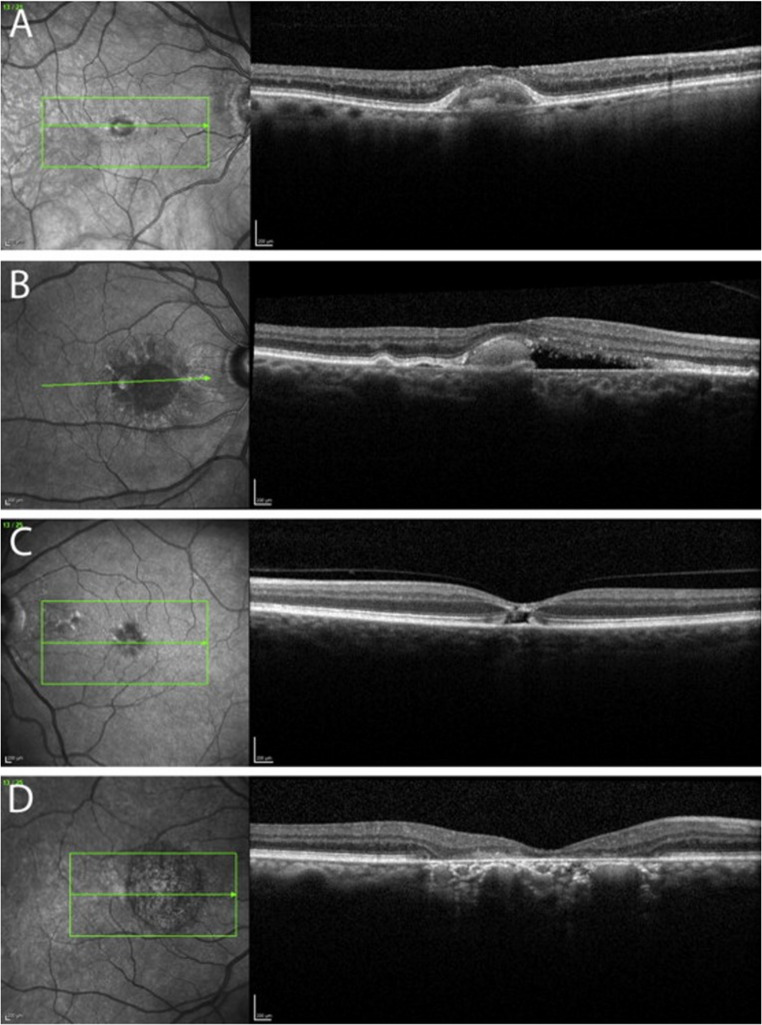
Stages of adult-onset foveomacular vitelliform dystrophy (AFVD) imaged by Spectral-domain optical coherence tomography (SD-OCT). (**A**), The vitelliform stage manifests as a homogenous subretinal hyper-reflective lesion (**B**) In the pseudo hypopyon stage, a hyperreflective area develops as a result of partial liquefaction of the lesion. (**C**) In the vitelliruptive stage, the hyper-reflective lesion flattens, most of the fluid is absorbed, and atrophy of the photoreceptors and retinal pigment epithelium ensues. (**D**) The atrophic stage is marked by complete atrophy of the photoreceptor outer and inner segments, and atrophy of the outer nuclear layer and retinal pigment epithelium (cRORA)

The onset of genetically proven AFVD is typically between ages 30 and 50. Like Best’s disease, the lesion in AFVD evolves through four different stages (vitelliform, pseudohypopion, vittelieruptive, and atrophic; Fig. 1). This process is accompanied by a loss of visual acuity [3]. In a series of 17 eyes by Greaves et al., over 40% of patients of one line in three years of follow-up [4]. In another series of 46 eyes followed over 16 months by Querques et al., mean visual acuity dropped from 0.32 LogMAR to 0.39 LogMAR [5]. 

Sporadic AFVD is the most common form and appears in the elderly population.

AFVD shares common features with age-related macular degeneration (AMD). An association between AFVD and different drusen types has been described, including cuticular drusen, sub-retinal drusenoid deposits and druesnoid pigment epithelial detachments [6–8]. In addition, Autosomal dominant AFVD cases can demonstrate the presence of drusen. These associations may reflect a shared pathogenic mechanism of RPE-photoreceptor complex malfunction [3]. 

While existing studies on AMD indicate minimal progression post-cataract surgery, the impacts and safety of cataract surgery in patients with coexisting AFVD remain unexplored [9, 10]. 

Given the similiarties in retinal involvement between AFVD and AMD, and the similarity in age presentation it is imperative to investigate whether cataract surgery in AFVD patients follows a similar trajectory as in AMD patients. Clarifying this relationship is crucial for enabling ophthalmologists to make evidence-based decisions regarding cataract management in AFVD patients, potentially enhancing visual outcomes and quality of life for there individuals. Thus, we aimed to evalute the outcomes and safety of cataract surgery in Adult-Onset Foveomacular-Vitelliform dystrophy (AFVD) patients.

## Methods

### Study design, patient selection, and data collection

This retrospective study was done in Hadassah Medical Center’s ophthalmology department (Jerusalem, Israel). The study was approved by the Institutional Review Board (IRB) and included a waiver of informed consent for the chart review (approval number HMO-20–0567). The study adhered to the tenets of the Declaration of Helsinki.

The electronic medical records of all patients with AFVD undergoing cataract surgery and followed at Hadassah Medical Center retina clinic from 2012 to 2021 were reviewed. AFVD patients above age 50 with one of the AFVD stages documented by OCT - vitelliform lesion smaller than 1 disc diameter; pseudohypopion; vittelieruptive; and atrophic- were included in the study based on previously described criteria [11]. All AFVD patients had genetic testing, and all were negative for the known mutations in PRPH2, IMPG1/2, and VMD2 [3]. 

The electronic medical records of patients with none-NV AMD and neovascular AMD undergoing cataract surgery and followed at Hadassah Medical Center retina clinic from 2012 to 2021 were reviewed and served as controls. Each control group’s sample size was selected to be approximately 2:1 larger relative to the AFVD group, and the patients were randomly selected. AMD patients presented at an age older than 50 years old with drusen or pigmentary changes (none-NV AMD), and signs of choroidal neovascularization (in NVAMD).

Patient records were reviewed from baseline to 1 (± 3 weeks), 3 (± 1 month), 6 (± 2 months), 12 (± 3 months) months post surgery and at last follow-up (documented only if greater than 15 months).

Demographics parameters included age, gender, laterality, AFVD stage, best-corrected visual acuity LogMAR (will be referred to as VA).

### Retinal imaging protocol and AFVD staging

SD-OCT images were taken using the Heidelberg spectralis (Heidelberg Engineering, Germany).

Each OCT scan had 61 B-scans per cube scan, with a width and height of 20^o^ in the horizontal and vertical directions. Scans were averaged using the automated real-time mode of the Spectralis system, with 30 frames per B-scan. The technical properties of OCT images and of the procedures used are described elsewhere [12]. 

AFVD staging relied on OCT scans as previously described [13] and devided AFVD to the vitelliform, pseudohypopion, vitellieruptive, and atrophic stages. Staging was carried out by two independent retina specialists (OS, LT).

### Study outcomes

Outcomes compared between AFVD and none and neovascular AMD included VA and optical coherence tomography (OCT) findings of new CNV or macular atrophy at 1 month,12 months, and last follow-up following cataract surgery.

### Statistical analysis

Statistical analysis was done using Graph-pad Prism software (version 9.0.0) and SPSS software (version 27, Chicago, IL). The normality of samples was tested using the Shapiro-Wilk test.Descriptive statistics, including means and standard deviations, were calculated for each parameter to summarize the data.The Kruskal-Wallis test was used to compare the AFVD, none-NV AMD, and neovascular AMD groups. The Mann-Whitney test was used to analyze the different time points between baseline and follow-up time points in each group. Chi-square test was used to compared proportions of AFVD stages at different time points. A P-value < 0.05 was considered significant.

### Unilateral sub-group analysis

This analysis added to strengthen the results and exclude misinterpretation due to possible inter-ocular similiarities within patients.

## Results

The study included 83 eyes (27 none-NV AMD eyes from 21 patients, 38 NVAMD eyes from 28 patients, 18 AFVD eyes from 11 patients). All eyes underwent uncomplicated phacoemulsification cataract surgery with intra-ocular lens implantation. Post-operative courses were uneventful with no prolonged corneal edema or cystoid macular edema. Follow-up was maintained for 1 month in all eyes and for 12 months in 13 none-NV AMD, 37 NVAMD, and 16 AFVD eyes. Extended follow up (greater than 15 months) was available for 20 none-NV AMD (34.10 ± 33.40 months), 38 NVAMD (64.20 ± 40.40 months), and 10 AFVD (10 eyes had BCVA but only 9 eyes had AFVD staging; 62.20 ± 39.30 months) eyes.

NVAMD patients’ baseline VA was significantly lower compared to the other groups (Table 1).

**Table 1:** Baseline patients characteristics

Group	AVFD	None-Neovascular AMD	NVAMD
Number of eyes	18	27	38
Follow up rate (months and percent of eyes):	1 M = 100% 3 M = 88.8% 6 M = 88.8% 12 M = 88.8% Last Follow-up = 55.5%	1 M = 100% 3 M = 62.9% 6 M = 51.8% 12 M = 48.1% Last Follow-up = 74.1%	1 M = 100% 3 M = 100% 6 M = 100% 12 M = 100% Last Follow-up = 100%
Age (years)	77.7 ± 5.7 (78; 71–87)	77.33 ± 10.18 (79; 61–92)	79.0 ± 7.05 (79.5; 59–90)
Males/Females (%)	38.9%/61.1%	59.3%/40.7%	39.5%/60.5%
AVFD stage (%)	Vitelliform = 12 (66.7%) Pseudohypopyon = 3 (16.65%) Vitellieruptive = 2 (11.1%) Atrophic = 1 (5.55%)	-	-
Baseline VA	0.60 ± 0.34 (0.5; 0.30–1.60)	0.65 ± 0.65 (0.49; 0.05–3.00.05.00)	1.10 ± 0.63 (0.9; 0.4–2.4)*

Continuous variables are presented by Mean ± standard deviation (median; minimum-maximum values)

*AVFD *Adult vitelliform foveal dystrophy; *AMD *Age related macular degeneration; *NVAMD *Neovascular AMD; *VA *Visual acuity, reported as LogMAR of best-corrected visual acuity; *P < 0.05

### Visual acuity improvement following cataract surgery

Detailed follow-up of AFVD eyes visual acuity and disease stage before and following cataract surgery is presented in Table 2.

**Table 2:** Demographic characteristics, laterality, visual acuity, and disease stage before and following cataract surgery in AFVD

Patient no.	Age at Surgery (years)	Gender	Laterality	Follow- up (Months)	BCVA LogMAR Pre-Op	BCVA LogMAR 1 Month Post-Op	BCVA LogMAR 12 Months Post-Op	BCVA LogMAR at Last Follow-up	AFVD Stage Pre-Op	AFVD Stage 12 Months Post-Op	AFVD Stage at Last Follow-up
1	78	M	RE	24	0.3	0.3	0.3	0.4	Vittelieruptive	Atrophic	Atrophic
	79		LE	13	0.3	0.4	0.4	NA	Atrophic	Atrophic	Atrophic
2	80	M	RE	34	0.5	0.3	0.3	0.3	Vitteliform	Vitteliform	Vitteliform
	80		LE	30	0.6	0.2	0.2	0.2	Vitteliform	Vitteliform	Vitteliform
3	87	F	RE	1	1.6	0.3	NA	NA	Vitteliform	NA	NA
4	74	F	RE	76	1	0.3	0.3	0.3	Vitteliform	Vitteliform	NA
	74		LE	72	1	0.16	0.16	0.16	Vitteliform	Vitteliform	NA
5	79	M	RE	1	0.7	0	NA	NA	Vitteliform	NA	NA
6	85	F	LE	12	0.3	0.16	0.16	NA	Vitteliform	Vitteliform	NA
7	71	F	RE	130	0.48	0.1	0.1	0.4	Pseudohypopion	Pseudohypopion	NA
	71		LE	130	0.48	0.1	0.1	0.9	Vitteliform	Vitteliform	NA
8	87	M	LE	12	0.5	0.3	0.4	NA	Vitteliform	Vitteliform	NA
	86		RE	18	0.5	0.4	0.4	0.4	Vitteliform	Vitteliform	NA
9	75	F	LE	72	0.4	0.1	0.1	0.5	Vitteliform	Vitteliform	Atrophic
	75		RE	36	0.4	0.16	0.4	1	Pseudohypopion	Vittelieruptive	Atrophic
10	78	F	RE	3	1	0.2	0.2	NA	Vitteliform	NA	Vitteliform
11	70	F	RE	9	0.3	0.3	0.3	NA	Pseudohypopion	Pseudohypopion	Pseudohypopion
	70		LE	12	0.5	0.4	0.4	NA	Vittelieruptive	Vittelieruptive	Vittelieruptive

*M *male, *F *female, *LE *left eye, *RE *right eye, *NA *not available, *AFVD *Adult Foveal Vitteliform Dystrophy

**Fig. 2:**
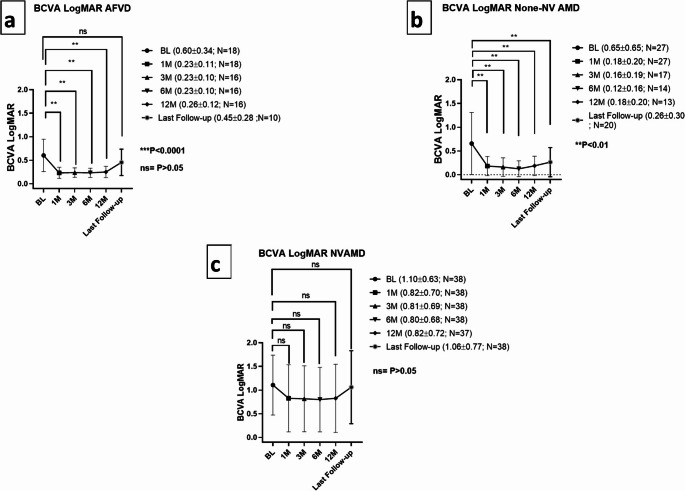
Visual acuity changes following cataract surgery in AFVD (*N* = 18), None-NV AMD (*N* = 27), and NVAMD (*N* = 38) patients. The mean follow-up times were 66.2 ± 39.30 months for AFVD, 34.10 ± 33.40 months for None-NV AMD, and 64.20 ± 40.40 months for NVAMD. N = number of eyes; M = months following surgery; BCVA LogMAR = LogMAR of best-corrected visual acuity; AFVD = Adult foveo-macular vitelliform dystrophy; AMD = Age related macular degeneration; None-NV AMD = None-neovascular age related macular degeneration; NVAMD = Neovascular age related macular degeneration *P-value < 0.05; *P-value < 0.01; **P-value < 0.001

VA of AFVD patients before surgery (0.60 ± 0.340) was statistically different compared with 1 month (0.23 ± 0.11) to 12 months post-operatively (0.26 ± 0.12); *P* < 0.0001 for both (Fig. 2a). 12 months post-operatively, no CNV occurred. The visual acuity deteriorated at last follow-up to 0.45 ± 0.28, and was no longer different from baseline (*P* = 0.21).

AFVD eyes with baseline vitelliform stage had a greater VA improvement after cataract surgery compared with eyes with more advanced AFVD stages (pseudohypopion, vitellieruptive or atrophic) at baseline (0.49 ± 0.12 and 0.10 ± 0.07, respectively; *P* = 0.035).

Significant improvements in VA following cataract surgery were shown in none-NV AMD patients as well (*P* < 0.01 for all timepoints Fig. 2b). In NVAMD patients, vision improved in a non-sigficant manner at 1–12 months post-op, and demonstrated a decline at last follow-up (*P* > 0.05 for all timepoints; Fig. 2c).

**Fig. 3:**
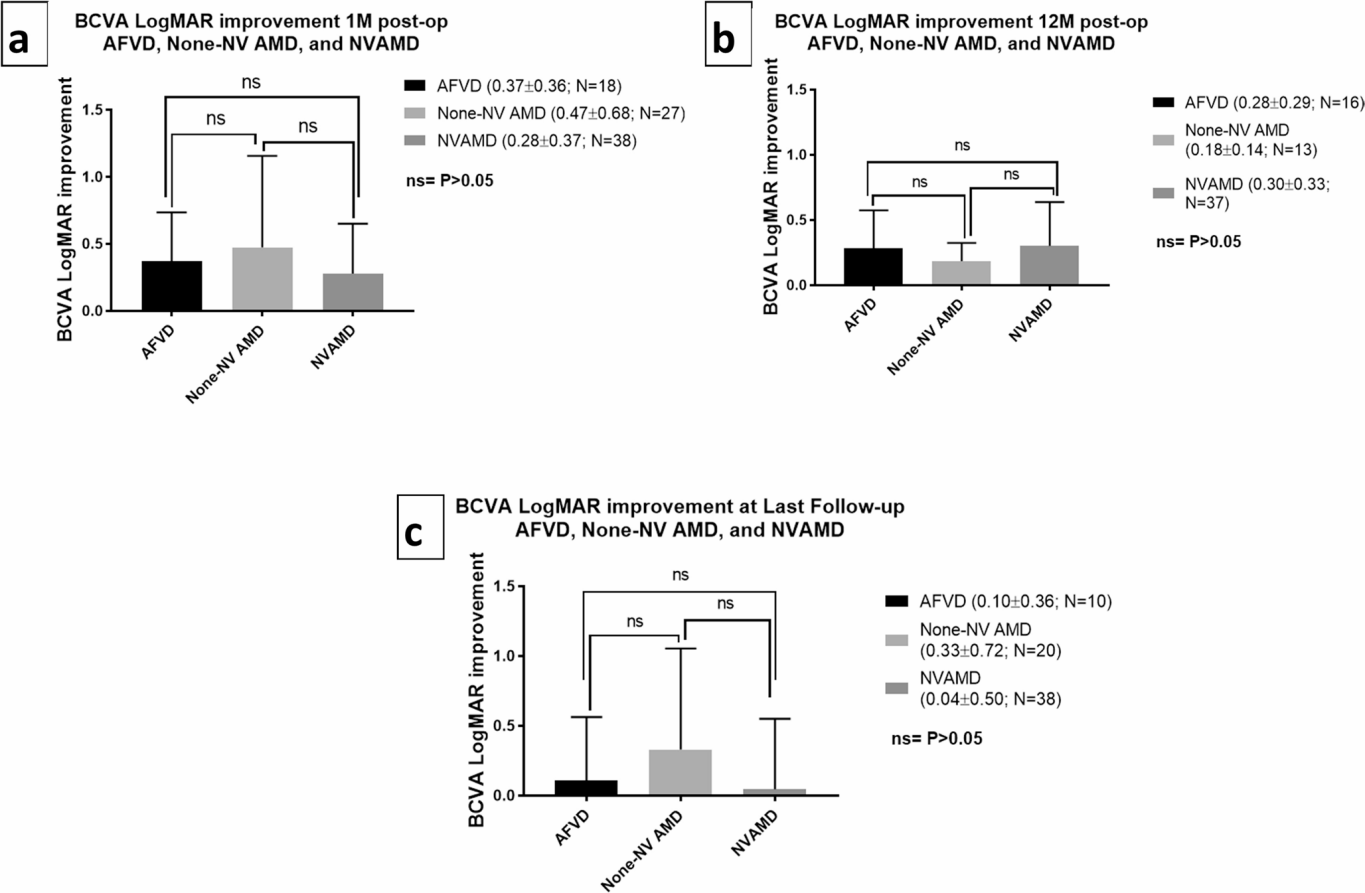
Comparison of visual acuity improvement 1 month, 12 months and at last extended follow-up post-cataract surgery in the different study groups. N = number of eyes; M = months following surgery; BCVA LogMAR = LogMAR of best-corrected visual acuity; AFVD = Adult foveo-macular vitelliform dystrophy; AMD = Age related macular degeneration; None-NV AMD = None-neovascular age related macular degeneration; NVAMD = Neovascular age related macular degeneration; ns = non-significant; *P-value < 0.05

No difference was found comparing the degree of VA improvement at one month (none-NV AMD = 0.47 ± 0.68; NVAMD = 0.28 ± 0.37, AFVD = 0.37 ± 0.36, *P* = 0.29), 12 months (none-NV AMD = 0.18 ± 0.14; NVAMD = 0.30 ± 0.33, AFVD = 0.28 ± 0.29, *P* = 0.47) and last follow-up after surgery (none-NV AMD = 0.33 ± 0.72; NVAMD = 0.04 ± 0.50, AFVD = 0.10 ± 0.36, *P* = 0.19) of none-NV AMD, NVAMD and AFVD (Fig. 3). In the none-NV AMD group, one eye developed CNV 10 months after surgery and one eye had worsening retinal atrophy one month post-surgery. In the NVAMD group, 9 eyes developed retinal atrophy at last follow up (89.50 ± 28.40 months for these 9 eyes).

### AFVD stage progression following cataract surgery

At baseline, 18 AFVD eyes were available, with 12 (66.60%) at the vitelliform stage. At twelve months, 15 AFVD eyes were available, with 9 (64.20%) at the vitelliform stage. At last follow-up (62.20 ± 39.30 months) 3 eyes (33% of 9 available eyes; *P* = 0.10 with Chi-Square test) were in the vitelliform stage, one eye was in the pseudohypopion stage, one eye was in the vitellieruptive stage, and 4 eyes were at the atrophic stage.

One eye showed actual progress from baseline vitellieruptive to the atrophic stage at 12 months.

**Fig. 4:**
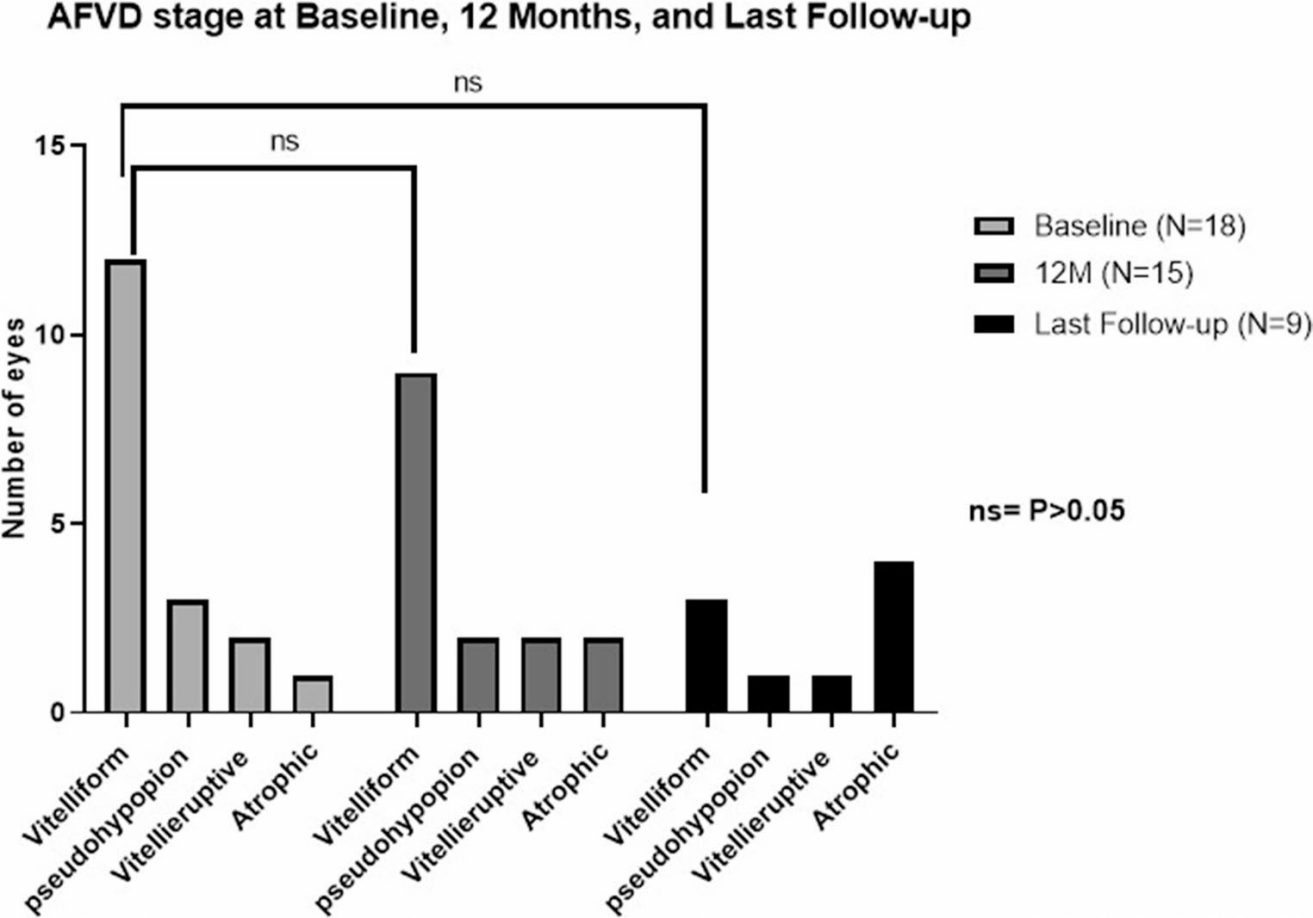
Comparison of AVFD stage before, 12 months and at last extended follow-up post-cataract surgery. At baseline, 18 AFVD eyes were available, with 12 (66.60%) at the vitelliform stage, and 1 (5.50%) at the atrophic stage. at twelve months, 14 AFVD eyes were available, with 9 (64.20%) at the vitelliform stage. At last follow-up (62.20 ± 39.30 months) 3 eyes (42% of 7 available eyes; *P* = 0.1 with Chi-Square test) 3 eyes were in the vitelliform stage and 4 eyes (57.10%; *P* = 0.02) at the atrophic stage N = number of eyes; M = months following surgery; AFVD = Adult foveo-macular vitelliform dystrophy

The percentage of eyes with atrophic AFVD increased from 5.50% (1/18) at baseline to 44.40% (4/9) at last follow-up (*P* = 0.10; Fig. 4). one eye with vitellieruptive stage at basline, one eye with pseudohypopyon stage at basline, and one eye with vitelliform stage at basline, showed actual progression to the atrophic stage from baseline to last follow-up.

### Unilateral sub-group analysis (available as supplementary material S1-S5)

In a unilateral sub-group analysis of 61 eyes (21 non-NV AMD, 29 NVAMD, 11 AFVD), results mirrored the overall sample. AFVD eyes showed significant visual acuity (VA) improvement from baseline (0.64 ± 0.40) to 1 month (0.22 ± 0.11) and 12 months (0.27 ± 0.10) post-cataract surgery (*P* < 0.05), with no CNV development. However, VA declined to 0.46 ± 0.26 at last follow-up, losing statistical significance from baseline (*P* = 0.30). AFVD eyes starting at the vitelliform stage tended towards better VA improvement post-surgery, though not significantly (*P* = 0.32). Non-NV AMD patients also had significant VA improvements. In NVAMD patients, VA improved non-significantly at 1, 3, and 12 months, but significantly at 6 months (*P* = 0.047), followed by a decline at last follow-up. Adverse events in the non-NV AMD group included eye developing CNV and another worsening retinal atrophy. Seven NVAMD eyes developed retinal atrophy at last follow-up (Figure S3). No significant differences in VA improvement were found when comparing the three groups (Figure S4).

AFVD stage progression showed non-significant trends (Figure S5).

One eye showed actual progress from baseline vitellieruptive to the atrophic stage at 12 months. Another eye showed actual progress from baseline pseudohypopyon to the atrophic stage at last follow up (36 months).

## Discussion

Following cataract surgery, mean VA improved significantly in eyes with AFVD in the first post-operative year, and the improvement was greater in the earlier AFVD stages- vitteliform and pseudohypopion. No CNV or acute new-onset retinal atrophy developed. No major changes in the stage of AFVD were noted in the first year. There was a visual decline and progression of atrophy following an extended mean follow-up of 41.5 months. There were no differences in VA improvement between the AFVD, none-NV AMD, and NVAMD groups at any timepoint.

AFVD and AMD have a described association and may coexist, implying a degree of shared pathogenic pathways involving RPE-photoreceptor complex malfunction [3, 6–8]. In light of the previously established safety of cataract surgery in AMD eyes [9, 10], none-NV AMD, and NVAMD were chosen to serve as the control groups in this study. It was impractical to select AFVD patients who did not undergo cataract surgery as controls due to the high prevalence of cataracts in this age group (most above 50 years), which may interfere with the attribution of visual acuity impairment to the stage and progression of retinal pathology.

To the best of our knowledge there are no previous studies on the safety of cataract surgery in AFVD.

VA improvement in AFVD following cataract surgery, as seen in the present work, is generally expected, as the integrity of photoreceptors is maintained until the latest stages of AFVD, characterized by decreasing photoreceptor function [14]. 

Our results demonstrate greater visual gain following cataract surgery in the vitteliform and pseudohypopion stages of AFVD, as opposed to the vittelieruptive and atrophic stages. Flattening of the vitelliform lesion with the emergence of outer retinal atrophy, RPE atrophy, and photoreceptor malfunction in the later stages of AFVD may account for this finding [3, 15]. 

In our study, progression of AFVD to the atrophic stages, accompanied by a visual decline from a mean BCVA of 0.23 1 month post-op to a mean BCVA LogMAR of 0.45 (approximately 2 snellen rows, to a final BCVA of 20/56), was seen only following 62.2 months of follow-up. Studies on AFVD’s natural history describe a slow estimated visual decline of 0.1 LogMAR over 30 months [16], with VA usually preserved above 20/50 in at least one eye [17]. In a large prospective cohort of 237 eyes with Adult vitteliform lesions, Chandra et al. revealed 21.9% showed macular atrophy at five years of follow-up [18]. This proportion is lower than 44.4% of eyes demonstrating atrophy at last follow-up of 66.2 months in the present work. Nonetheless, only 9 of 18 eyes had available morphologic staging at last follow-up in the current study. It should also be noted that only one of the AFVD eyes that progressed to the atrophic stage was in the vitteliform stage at baseline, indicating that progression of AFVD was already active before cataract surgery. Together, these observations generally imply that AFVD progression seen in some of the eyes following cataract surgery in our cohort is the result of the natural history of the disease, which includes decreased outer segement uptake due to impaired RPE phagocytosis [3]. Altogether, these findings support the safety of cataract surgery in AFVD.

Our study has several limitations. First, a relatively small sample size of AFVD eyes was included; a larger sample may have improved the precision of our calculations and discovered additional significant findings. Second, bilateral cases were included, which may cause errors in the results due to similarities between the two eyes of the same patient and compounding of the data. However, a subgroup analysis of unilateral cases was done, and results mirrored the overall sample (available as supplementary materials S1, S2, S3, S4, S5).

Third, only 9 of 18 AFVD eyes in our study had available staging for a period longer than 12 months; longer follow-up might aid in revealing the long-term effects of cataract surgery on the progression of AFVD. Fourth, patients treated at a tertiary center, as in the present study, usually present with more advanced ocular pathology; however, most AFVD eyes in our cohort presented at the initial vitelliform stage.

Fifth, cataract severity data was unavailable. However, the lack of intra- or post-operative complications (like corneal edema or cystoid macular edema) suggests severity didn’t significantly impact visual improvement differences across groups. Lastly, quantification of ellipsoid zone integrity and microperimetry results before and after cataract surgery would augment our findings.

To conclude, our study reports the outcomes of cataract surgery in AVFD eyes for the first time, to the best of our knowledge. Significant visual acuity improvements were seen with no significant complications. These findings indicate the safety and efficacy of cataract surgery in AFVD eyes.

Further research with larger sample numbers, prospective study design, longer follow-up, ellipsoid zone integrity quantification, and micro-perimetry usage is required to clarify the clinical implications of our results.

## Supplementary Information

Below is the link to the electronic supplementary material.

Supplementary File 1 (DOCX 571 KB)

## Data Availability

Clinical data reported in this work are available upon request from the corresponding author.
