# Supplementary material for: Cataract surgery in eyes with adult-onset foveomacular-vitelliform dystrophy
Source: Graefes Arch Clin Exp Ophthalmol. 2025 Nov 3;264(2):355–64. doi: 10.1007/s00417-025-07018-x (PMC12923482; doi:10.1007/s00417-025-07018-x)
Supplement: Supplementary file 1 — (DOCX 571 KB) [file 417_2025_7018_MOESM1_ESM.docx]

**Cataract Surgery in Eyes with Adult-Onset Foveomacular-Vitelliform Dystrophy (Unilateral Sub-group Analysis; Supplementary material)**

Or Shmueli, MD (1), Tomer Batash, MD (1), Itay Nitzan, MD (1), Itay Chowers, MD (1), Liran Tiosano, MD (1)

**Correspondence**:

Or Shmueli, MD, Ophthalmology Department, Hadassah Medical Center, Faculty of Medicine, Hebrew University, Jerusalem 91120, POB 12000, Israel.

Tel.: ‏+972547631035.

E-mail address: [Orchuk86@gmail.com](mailto:Orchuk86@gmail.com)

**Table S1.** **Baseline** **Patients Characteristics for Unilateral Sub-group Analysis**

| **NVAMD** | **None-Neovascular AMD** | **AVFD** | **Group** |
| --- | --- | --- | --- |
| 29 | 21 | 11 | **Number of eyes** |
| 1M= 100%  3M= 100%  6M= 100%  12M= 96.5%  Last Follow-up= 100% | 1M= 100%  3M= 57.1%  6M= 47.6%  12M= 47.6%  Last Follow-up= 71.4% | 1M= 100%  3M= 81.8%  6M= 81.8%  12M= 72.7%  Last Follow-up= 54.5% | **Follow up rate (months and percent of eyes):** |
| 78.6±7.4 (79; 59-90) | 77.9±10.0 (79; 61-92) | 78.4±5.7 (78; 70-87) | **Age (years)** |
| 58.6%/41.4% | 61.9%/38.1% | 36.4%/63.6% | **Males/Females (%)** |
| - | - | Vitelliform=7 (63.6%)  Pseudohypopyon=3 (27.2%)  Vitellieruptive=1 (9.1%)  Atrophic=0 (0%) | **AVFD stage (%)** |
| 1.14±0.60 (0.9; 0.4-2.3)***** | 0.59±0.49 (0.49; 0.05-1.95) | 0.64±0.40 (0.5; 0.30-1.60) | **Baseline VA** |

Continuous variables are presented by Mean±standard deviation (median; minimum-maximum values).

AVFD=Adult vitelliform foveal dystrophy; AMD= Age related macular degeneration; NVAMD= Neovascular AMD; VA= Visual acuity, reported as LogMAR of best-corrected visual acuity; *P<0.05

**Table 2S: Demographic characteristics, laterality, visual acuity, and disease stage before and following cataract surgery in AFVD for unilateral cases**

| **AFVD Stage at Last Follow-up** | **AFVD Stage 12 Months**  **Post-Op** | **AFVD Stage Pre-Op** | **BCVA LogMAR at Last Follow-up** | **BCVA LogMAR 12 Months**  **Post-Op** | **BCVA LogMAR 1 Month**  **Post-Op** | **BCVA LogMAR Pre-Op** | **Follow- up (Months)** | **Laterality** | **Gender** | **Age at Surgery (years)** | **Patient no.** |
| --- | --- | --- | --- | --- | --- | --- | --- | --- | --- | --- | --- |
| atrophic | atrophic | Vittelieruptive | 0.4 | 0.3 | 0.3 | 0.3 | 24 | RE | M | 78 | **1** |
| Vitteliform | Vitteliform | Vitteliform | 0.3 | 0.3 | 0.3 | 0.5 | 34 | RE | M | 80 | **2** |
| NA | NA | Vitteliform | NA | NA | 0.3 | 1.6 | 1 | RE | F | 87 | **3** |
| NA | Vitteliform | Vitteliform | 0.3 | 0.3 | 0.3 | 1 | 76 | RE | F | 74 | **4** |
| NA | Vitteliform | Vitteliform | NA | NA | 0 | 0.7 | 1 | RE | M | 79 | **5** |
| NA | Vitteliform | Vitteliform | NA | 0.16 | 0.16 | 0.3 | 12 | LE | F | 85 | **6** |
| NA | Pseudohypopion | Pseudohypopion | 0.4 | 0.1 | 0.1 | 0.48 | 130 | RE | F | 71 | **7** |
| NA | Vitteliform | Vitteliform | 0.4 | 0.3 | 0.4 | 0.5 | 18 | RE | M | 86 | **8** |
| atrophic | Vittelieruptive | Pseudohypopion | 1 | 0.4 | 0.16 | 0.4 | 36 | RE | F | 75 | **9** |
| Vitteliform | NA | Vitteliform | NA | 0.2 | 0.2 | 1 | 3 | RE | F | 78 | **10** |
| Pseudohypopion | Pseudohypopion | Pseudohypopion | NA | NA | 0.3 | 0.3 | 9 | RE | F | 70 | **11** |

M: male, F: female, LE: left eye, RE: right eye, NA: not available, AFVD: Adult Foveal Vitteliform Dystrophy

**Figure S3. Visual acuity changes following cataract surgery in AFVD (N=11), None-NV AMD (N=21), and NVAMD (N=29) patients**

**(one eye per patient).**


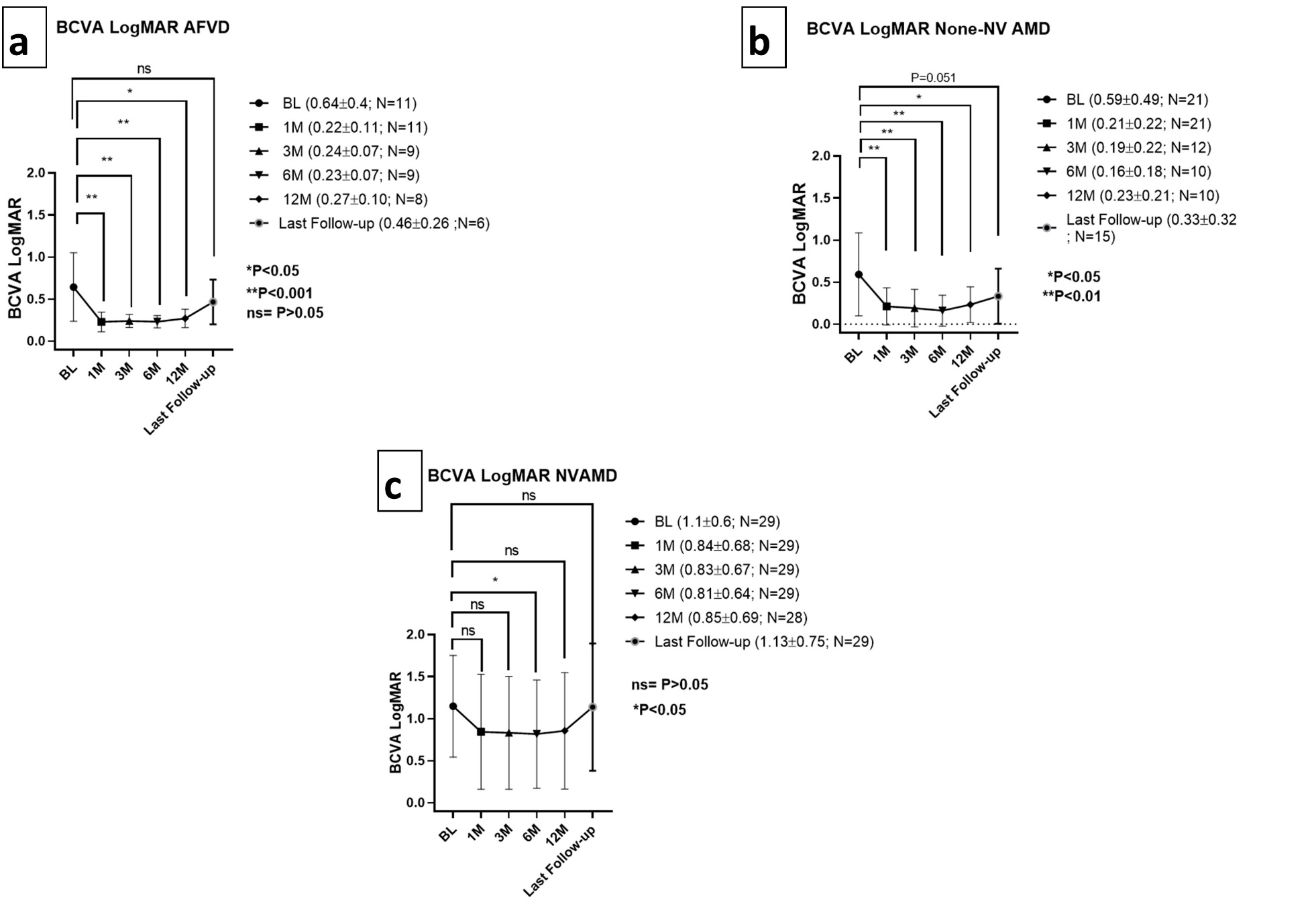


N=number of eyes; M=months following surgery; BCVA LogMAR= LogMAR of best-corrected visual acuity; AFVD =Adult foveo-macular vitelliform dystrophy; AMD= Age related macular degeneration; None-NV AMD= None-neovascular age related macular degeneration; NVAMD= Neovascular age related macular degeneration *P-value<0.05; **P-value<0.01; ns=non-significant

**Figure S4. Comparison of visual acuity improvement 1 month, 12 months and at last extended follow-up post-cataract surgery in the different study groups (one eye per patient).**


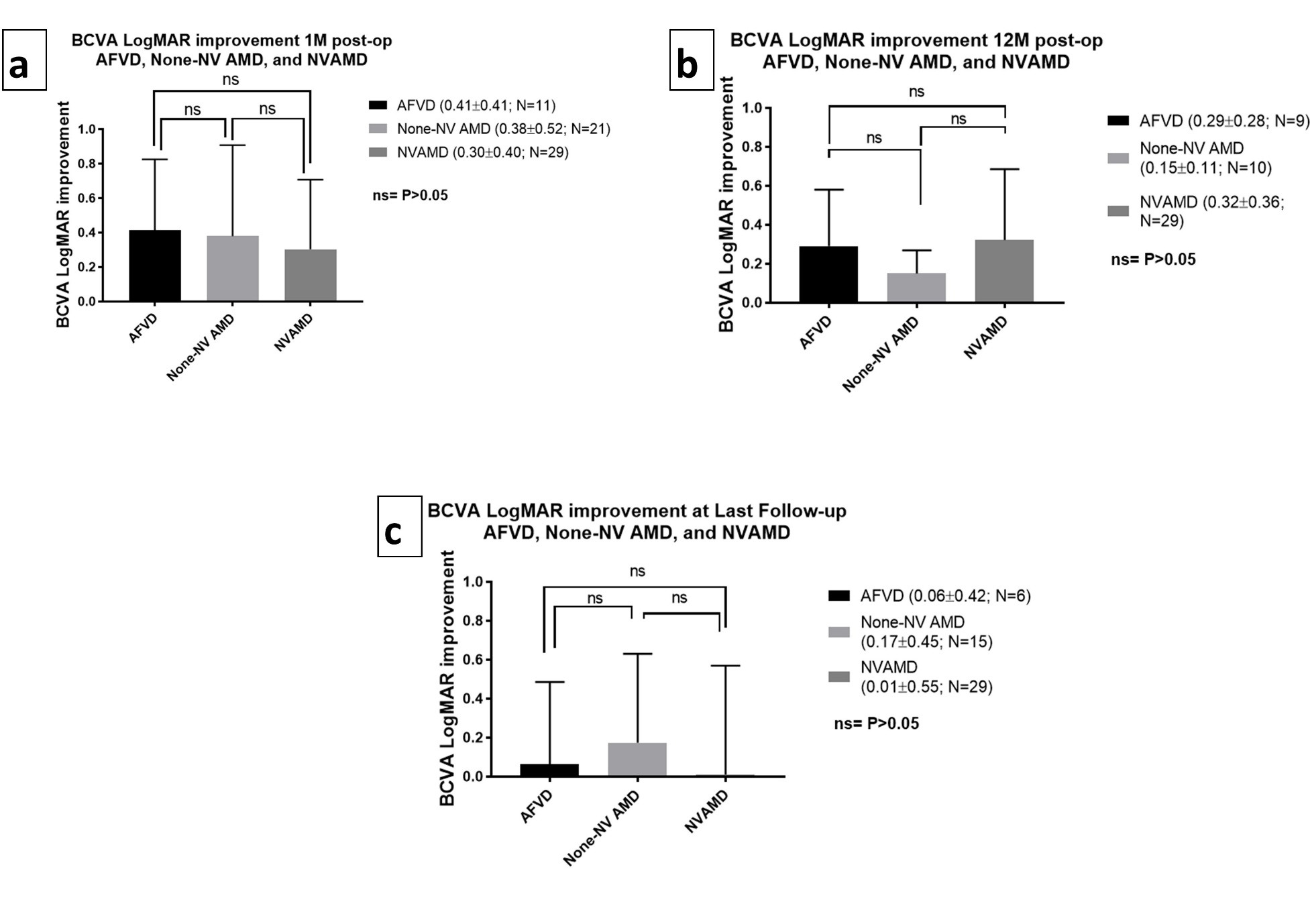


N=number of eyes; M=months following surgery; BCVA LogMAR= LogMAR of best-corrected visual acuity; AFVD =Adult foveo-macular vitelliform dystrophy; AMD= Age related macular degeneration; None-NV AMD= None-neovascular age related macular degeneration; NVAMD= Neovascular age related macular degeneration; ns=non-significant

**Figure S5. Comparison of AVFD stage before, 12 months and at last extended follow-up post-cataract surgery (one eye per patient).**


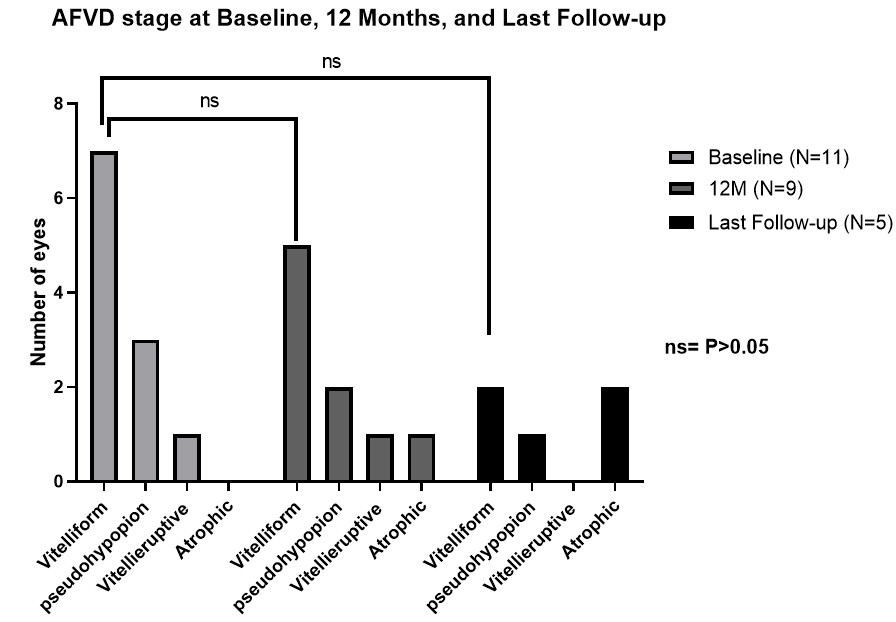


N=number of eyes; M=months following surgery; AFVD =Adult foveo-macular vitelliform dystrophy
